# Identification of the optic recess region as a morphogenetic entity in the zebrafish forebrain

**DOI:** 10.1038/srep08738

**Published:** 2015-03-04

**Authors:** Pierre Affaticati, Kei Yamamoto, Barbara Rizzi, Charlotte Bureau, Nadine Peyriéras, Catherine Pasqualini, Michaël Demarque, Philippe Vernier

**Affiliations:** 1 TEFOR Core Facility, Paris-Saclay Institute of Neuroscience (UMR9197), CNRS Université Paris-Sud, 91190 Gif-sur-Yvette, France; 2 Paris-Saclay Institute of Neuroscience (UMR9197), CNRS Université Paris-Sud, 91190 Gif-sur-Yvette, France; 3 BioEmergences (USR3695), CNRS, 91190 Gif-sur-Yvette, France

## Abstract

Regionalization is a critical, highly conserved step in the development of the vertebrate brain. Discrepancies exist in how regionalization of the anterior vertebrate forebrain is conceived since the “preoptic area” is proposed to be a part of the telencephalon in tetrapods but not in teleost fish. To gain insight into this complex morphogenesis, formation of the anterior forebrain was analyzed in 3D over time in zebrafish embryos, combining visualization of proliferation and differentiation markers, with that of developmental genes. We found that the region containing the preoptic area behaves as a coherent morphogenetic entity, organized around the optic recess and located between telencephalon and hypothalamus. This optic recess region (ORR) makes clear borders with its neighbor areas and expresses a specific set of genes (*dlx2a*, *sim1a* and *otpb*). We thus propose that the anterior forebrain (secondary prosencephalon) in teleosts contains three morphogenetic entities (telencephalon, ORR and hypothalamus), instead of two (telencephalon and hypothalamus). The ORR in teleosts could correspond to “telencephalic stalk area” and “alar hypothalamus” in tetrapods, resolving current inconsistencies in the comparison of basal forebrain among vertebrates.

The ordered development of the vertebrate brain integrates several concomitant events: dynamic morphogenetic processes, controlled proliferation and maturation of neural progenitor cells, and positional information specifying cell fate locally or at long range^1^,^2^,^3^,^4^. The spatial control of neuronal specification depends on the expression of a specific set of genes within discrete territories of the neural tube, which will become the main anatomic divisions of the brain^3^,^5^,^6^. This widely accepted concept of brain regionalization, by which the neural tube is patterned into distinct topological domains, has been refined over the years, notably with the introduction of neuromeric models of brain patterning^7^,^8^,^9^. The neuromeres are defined as transversal divisions of the neural tube. They are morphogenetic entities with a transiently recognizable morphology and they are characterized individually by different molecular identities or “genoarchitectures”^10^.

Neuromeres are well established in the rhombencephalon (rhombomeres), where segmentation shaped by specific genetic and cellular mechanisms is clearly observed^9^,^11^. In contrast, the situation in the prosencephalon, the most anterior of the three vesicles of the developing brain, remains more controversial, largely due to the extensive morphological changes affecting this territory during development. Fate mapping studies have suggested that the prosencephalic region of the neural plate comprises the primordia of the telencephalon, eye field, hypothalamus, and diencephalon^6^,^12^,^13^,^14^,^15^. A significant anterior-posterior rearrangement occurs in the forebrain during the formation of the neural tube. Studies in zebrafish have shown that the hypothalamic anlage is relocated by forward subduction below the presumptive telencephalon, favoring the lateral positioning of a large part of the eye field^15^,^16^.

Despite difficulties presented by the extensive changes in morphology, neuromeres were also proposed in the prosencephalon (prosomeres) based on developmental gene expression and morphological hallmarks^7^,^8^,^17^. In the current prosomeric model, the forebrain is divided into the caudal diencephalon and the rostral secondary prosencephalon^18^, the latter containing the future telencephalon, hypothalamus, and the retina. In the secondary prosencephalon, transversal segmentation has been difficult to recognize due to its complex morphology^8^. A main longitudinal axis through the neural tube is the boundary between alar and basal plates, which has been proposed to extend up to the most rostral end of the neural tube^19^. In the secondary prosencephalon, the hypothalamus is considered to be subdivided into alar and basal domains, and the telencephalon to be derived from the roof plate of the anterior neural tube^8^,^18^.

These general events of regionalization are thought to be conserved among different vertebrate groups, notably at the developmental time point referred to as the “phylotypic period” during which progenitor cells acquire a positional identity. Thus the comparison of gene expression patterns at development stages among species have often been interpreted within the framework of the prosomeric model^20^,^21^,^22^,^23^. When analyzed in detail however, there exist a number of discrepancies observed among species. Most notably, while the preoptic area has been proposed to be a part of the telencephalon in tetrapods^18^,^23^,^24^, it is identified as a region distinct from the telencephalon and the hypothalamus in teleosts^25^. Although the preoptic area occupies a large part of the anterior forebrain in adult teleosts, the development of this region has not been well studied.

To verify whether there is a distinct morphogenetic entity giving rise to the preoptic area in the teleost forebrain, we examined the course of neural differentiation of this region in zebrafish embryos based on the location and shape of the brain ventricles, progenitor cell maturation, and the expression of developmental genes. The analysis was performed systematically in three dimensions (3D) and at three developmental stages. We started our analysis at 48 hpf, when the initial wave of neurogenesis is achieved. To better understand the dynamics leading to this configuration, we extended our analysis backwards to earlier developmental stages: 30 hpf when brain ventricles are inflated^26^, and 24 hpf when brain ventricles are just opening and the initial scaffold of axon bundles is forming.

We found that the region containing the zebrafish preoptic area behaves as a morphogenetic entity, distinct from the telencephalon and the hypothalamus. As it is organized around the optic ventricular recess, we named this new morphogenetic entity the “optic recess region” (ORR). We compare our data with the current prosomeric model and discuss possible homology with tetrapods.

## Results

Anatomical descriptions are oriented with reference to the body axis and neuraxis, following Herget et al. (2014)^27^. We use terms “rostral” “caudal” “dorsal” “ventral” to refer the axial system co-linear to body parts. Generally the images shown in figures follow this body axis. We use terms “anterior” and “posterior” to take into account the temporal changes of the orientation of axes resulting from the curvature of the neuraxis. For instance, in 24 hpf embryos, the telencephalon is located rostrally and the hypothalamus is located caudally relative to each other, but they both exhibit an anterior topology in the prosencephalon at the stages we examined.

### 3D analysis of ventricular morphology in the 48 hpf zebrafish forebrain reveals the shape of the optic recess

**Figure 1:**
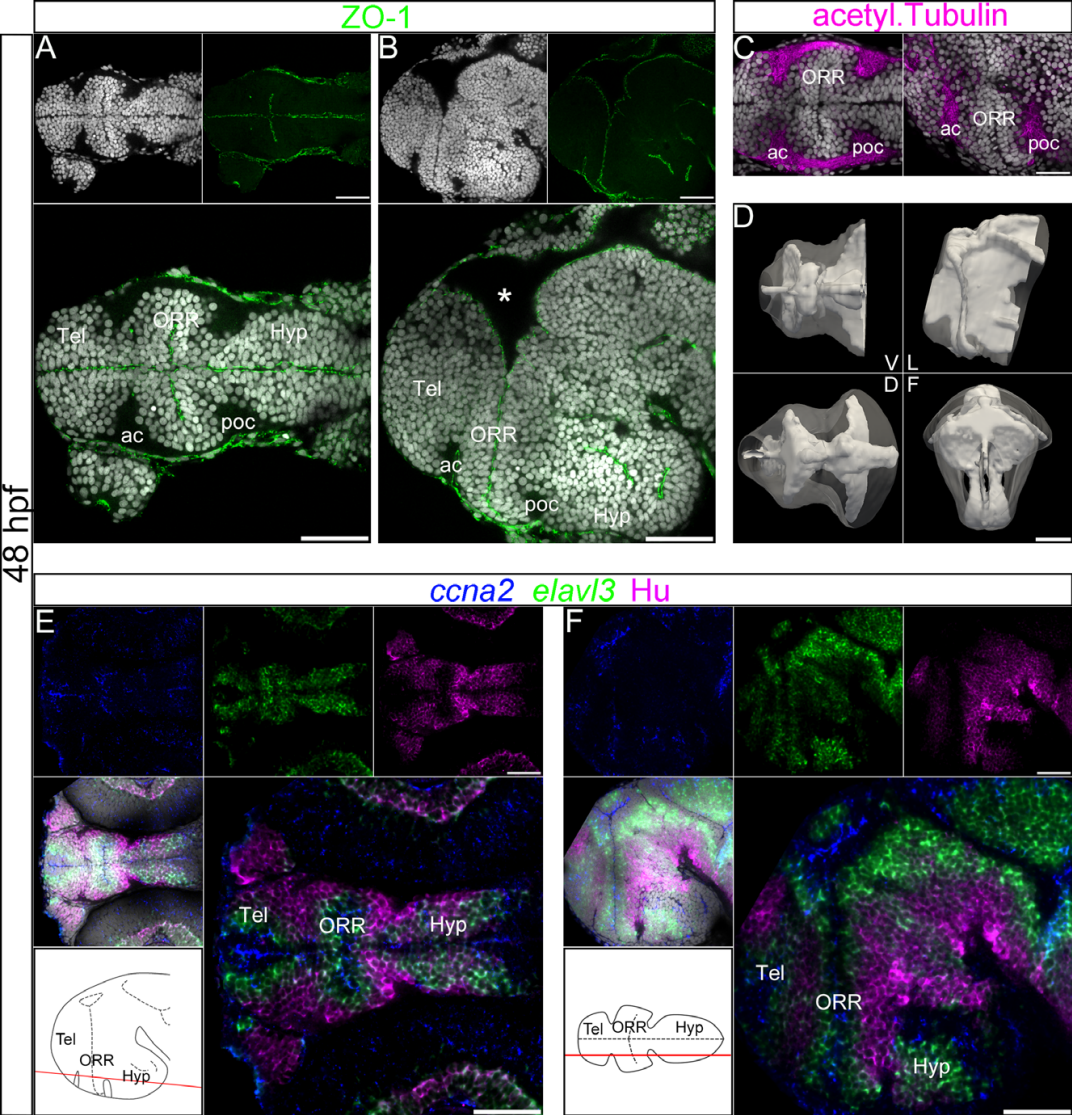
Ventricular organization and the centrifugal gradient of neurogenesis at 48 hpf. (A–C): Single confocal plane of a 48 hpf embryonic forebrain stained with DAPI (gray), immunolabeled for ZO-1 (A and B) or acetylated α-tubulin (C), in ventral (A and left panel in C) and lateral (B and right panel in C) views. The telencephalon (Tel), the optic recess region (ORR), and the hypothalamus (Hyp) form three distinct cellular regions in the secondary prosencephalon. The ORR is bordered by the dense fiber bundles, the anterior commissure (ac) and post-optic commissure (poc). The asterisk (*) in (B) (lateral view) indicates the enlargement of the dorsal ventricular lumen corresponding to the anterior intraencephalic sulcus (AIS). (D): Surface rendering of the shape of ventricle (white, in opacity), reconstructed from ZO-1 immunolabeling, overlapped on the shape of the brain (white, in transparency), reconstructed from DAPI staining. V = ventral, D = dorsal, L = lateral and F = frontal views. The 3D representation of both ventricle and brain facilitates the visualization of the convoluted ventricular organization in the forebrain. (E–F): Single confocal plane of a 48 hpf embryonic forebrain labeled for the neurogenic markers *ccna2*, *elavl3* and HuC/D in ventral (E) and lateral (F) views. Bottom left drawings show the level of corresponding optical planes. *ccna2*-positive proliferative cells are concentrated around the ventricular zones, while *elavl3*-positive differentiating and HuC/D-positive differentiated cells are located at the periphery of the neural tube. Scale bars = 50 μm.

In the developing brain, neurogenesis is organized around the ventricle in a centrifugal manner^2^,^28^. Thus, to accurately analyze the morphogenesis of brain structures, we first extracted the morphology of the ventricles of the zebrafish secondary prosencephalon. To visualize the ventricular walls and to collect data suitable for reconstructing the 3D shape of the ventricular system, we used immunodetection of ZO-1, a marker of the tight junctions labeling the apical side of neuroepithelial cells lining the ventricles^29^. In addition, the general organization of cells and their nuclei within the neural parenchyma was analyzed by a nuclear counterstain (DAPI). In a ventral view, the ventricle at 48 hpf takes the shape of a latin cross (Fig. 1A) with the lateral extension of the ventricles originating at the level of the optic stalk. This lateral extension of the ventricle spreads over the whole dorso-ventral thickness of the anterior neural tube (Figs. 1A–C and S1). From a lateral view, it displays an inverted S-shape bent rostrally in its dorsal extension and bent caudally in its large ventral extension, with an inflexion point located roughly half-way through its dorso-ventral expansion. The dorsal ventricular lumen is especially enlarged (asterisk in Fig. 1B), which corresponds to the anterior intraencephalic sulcus (AIS) lying between the telencephalon and the diencephalon^30^. In contrast, no such enlargement exists ventrally. The narrowing of the lateral ventricle ventrally is clearly highlighted in the frontal view (Fig. 1D: frontal view and Supplementary Material 1). We refer to this ventral part of the lateral ventricular extension as the optic recess.

More caudally, in the hypothalamus, two other recesses extend laterally. The rostral one, the lateral recess, is oriented roughly along the horizontal plane of the forebrain, while the posterior recess is oriented along the dorso-ventral plane (Fig. S1B,C). Thus, we systematically analyzed anatomical data with regard to the complex morphology of the ventricle through three orthogonal views simultaneously, scrolling up and down each view to encompass the whole shape of the brain.

It is worth noting that DAPI staining itself provides relevant information regarding the position of the ventricles, as the ZO-1 staining systematically coincides with a clear gap between cell nuclei (Fig. 1A and B). Moreover, the nuclei of the cells lining the ventricles are elongated and densely packed, while those of the periphery are rounded and more sparsely distributed. These morphological and cytological features can be used in subsequent analyses to deduce the position of the ventricle from cell morphology, even in the absence of ZO-1 staining.

### Three distinct cell masses are juxtaposed in the zebrafish ventral secondary prosencephalon

The general distribution of cell nuclei with reference to the ventricular morphology clearly identified three distinct cellular masses, each centered on a ventricular extension (Fig. 1A, bottom panel). The rostral-most cellular mass corresponds to the telencephalic region and develops around the rostral medial ventricle (telencephalic ventricle). The caudal-most cellular mass corresponds to the hypothalamic region and develops around the caudal part of the medial ventricle and the lateral and posterior recesses (Figs. 1B and S1). The region situated between the telencephalic and hypothalamic regions is symmetrically organized around the optic recess, which is oriented perpendicularly to the medial ventricle; thus we have termed it the optic recess region (ORR).

In the developing brain, neuronal fibers form bundles and establish other important and useful anatomical landmarks that can be visualized by the expression of acetylated α-tubulin (Figs. 1C and S1E). These bundles are located at the periphery of the brain, with very few tracts in the medial region. Two of these bundles, the anterior commissure (ac) and the postoptic commissure (poc), are flanking the ORR, further highlighting the three regions described in the previous paragraph (figs. 1C and S1E). Additionally, GFAP-labeled processes of radial glia appear more densely packed at the rostral and caudal limits of the ORR domain, abutting the telencephalon and hypothalamus respectively. This higher density of glia and their processes is easily visible on ventral horizontal sections, and corresponds to the location of the two commissures (Fig. S1E). Furthermore, based on the position of these commissures with regard to the brain, it can be deduced that the ORR corresponds to the region identified as the preoptic area in the mature zebrafish brain.

### Centrifugal organization of proliferation and differentiation from the ventricle to the periphery in the zebrafish secondary prosencephalon

To study the dynamics of cell maturation, from proliferation to differentiation, in relation with ventricular morphology, we simultaneously analyzed a series of progressive maturation markers: cyclin A2 (*ccna2*) transcripts, expressed at G1/S and G2/M transitions, were used to label proliferation, *elavl3*/HuC transcripts to label differentiating neurons, and HuC/D immunolabeling to label differentiated neurons. The translation of the HuC/D protein is delayed from the transcription of its corresponding *elavl3* mRNA, enabling a convenient spatial visualization of the time course of neuronal maturation.

At 48 hpf, proliferating cells (*ccna2*-positive) are restricted to a narrow band bordering the ventricular wall (Fig. 1E and F). Exclusively in this *ccna2*-positive zone, DAPI staining shows nuclei characteristic of dividing cells, with an elongated shape, sometimes forming doublets, and with dense/bright staining compared to the uniformly stained, non-dividing nuclei (Fig. S1D). Differentiating cells (*elavl3*-positive, HuC/D-negative) are located in a domain adjacent and lateral to proliferating cells. Differentiated cells (HuC/D-positive) are detected in the marginal zone of the neural tube, partially overlapping with the outermost *elavl3*-positive rows of cells. The comparison of the localization territories of these markers fits well with the general centrifugal organization of neurogenesis within the neural tube. Most interestingly, this pattern of organization is consistent in the telencephalic, ORR and hypothalamic regions. Hence, these three regions are generated from and around the telencephalic ventricle, optic recess, and hypothalamic ventricle respectively, each forming a distinct morphogenetic entity.

### Analysis of ventricular morphology and cell maturation at 30 hpf and 24 hpf confirms that three morphogenetic entities compose the ventral secondary prosencephalon

In order to better understand how these three regions take shape, we looked at earlier stages of development to more precisely define the relationship between cell proliferation/differentiation and morphogenesis of the anterior neural tube over time. To this aim, we used the same approach as at 48 hpf, but respectively at the end (30 hpf) and at the beginning (24 hpf) of the ventricle expansion process.

**Figure 2:**
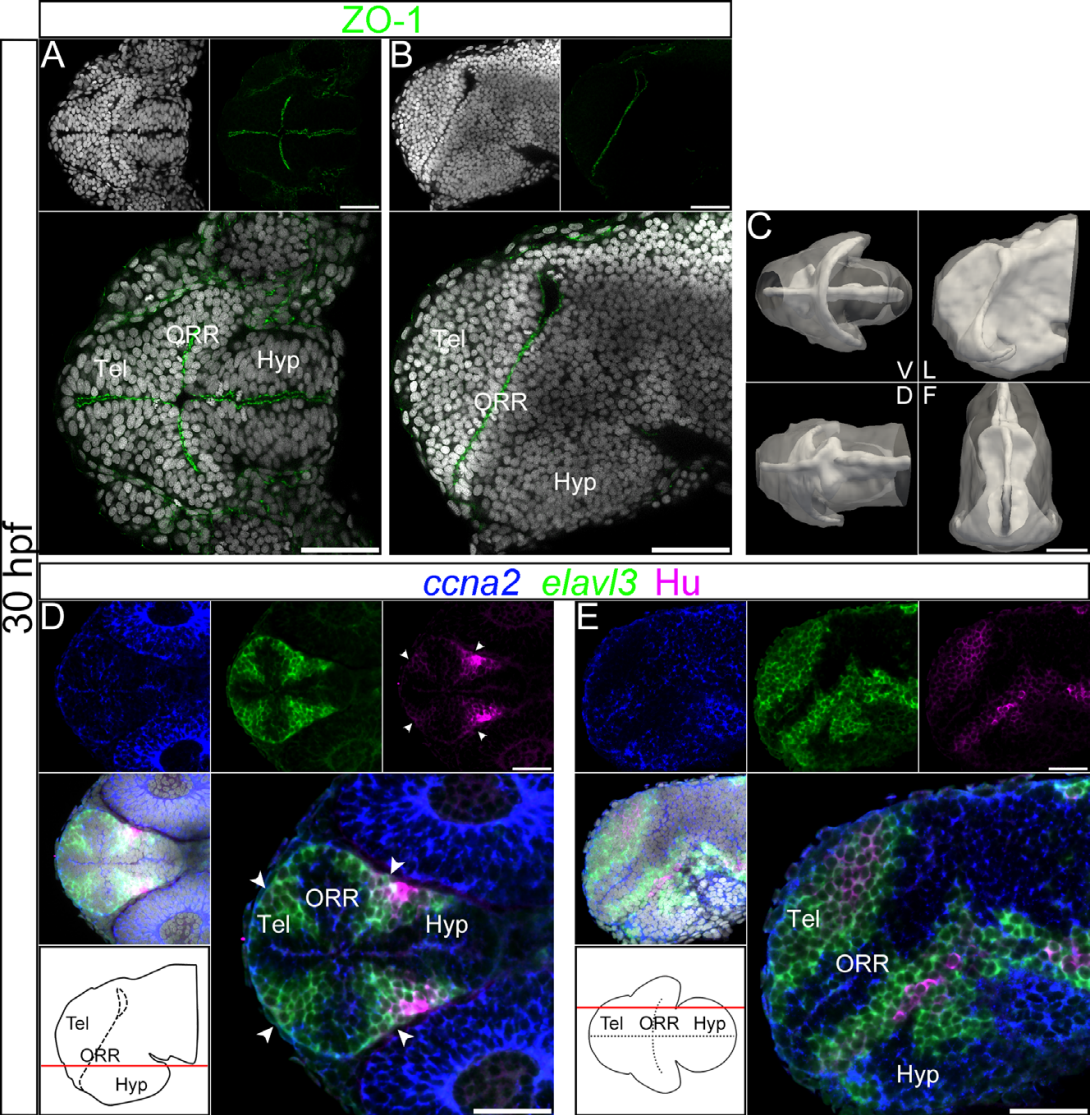
Ventricular organization and the centrifugal gradient of neurogenesis at 30 hpf. (A–B): Single confocal plane of a 30 hpf embryonic forebrain stained with DAPI (gray) and immunolabeled for ZO-1, in ventral (A) and lateral (B) views. (C): Surface rendering of the shape of ventricle (white, in opacity), reconstructed from ZO-1 immunolabeling, overlapped on the shape of the brain (white, in transparency), reconstructed from DAPI staining. V = ventral, D = dorsal, L = lateral and F = frontal views. At 30 hpf the organization of the ventricular system is less complex than at 48 hpf. The frontal view displays a characteristic keyhole shape of the ventricle, with a larger lateral expansion of the ventral part compared to the dorsal part. (D–E): Single confocal plane of a 30 hpf embryonic forebrain labeled with the neurogenic markers *ccna2*, *elavl3* and HuC/D, in ventral (D) and lateral (E) views. Bottom left drawings show the level of the corresponding optical plane. The *ccna2* staining is located around the ventricular walls but in wider territories than at 48 hpf. The *elavl3* staining is detected adjacent to the *ccna2* staining, also in wider territories than at 48 hpf. Accordingly, much fewer HuC/D-positive cells are detected than at 48 hpf, and they are all distributed at the periphery of the neural tube. The arrowheads in D indicate Hu-expressing cells located at the boundaries of telencephalon/ORR and ORR/hypothalamus. Scale bars = 50 μm.

At 30 hpf, the telencephalon, ORR and hypothalamus are already clear as distinct morphological masses, however, the organization of the ventricle is more simple than at 48 hpf (Fig. 2, for a detailed visualization of ventricular morphology see Fig. S2 and Supplementary Material 1). Specifically, the enlargement of the lumen dorsal to the optic recess is much smaller and the hypothalamic recesses are not yet apparent, but the “latin cross” formed by the intersection of the medial ventricle and the optic recess is present. When compared to 48 hpf, proliferating zones labeled with *ccna2* are wider (nonetheless mitotic cells are found only along the ventricular wall, Fig. S2C) and the territories labeled by *elavl3* and Hu-protein are smaller (Fig. 2D and E). At the border between the ORR and the hypothalamus/diencephalon, Hu-positive differentiated cells (Fig. 2D, arrow heads) are clearly flanked by *elavl3* on both sides.

**Figure 3:**
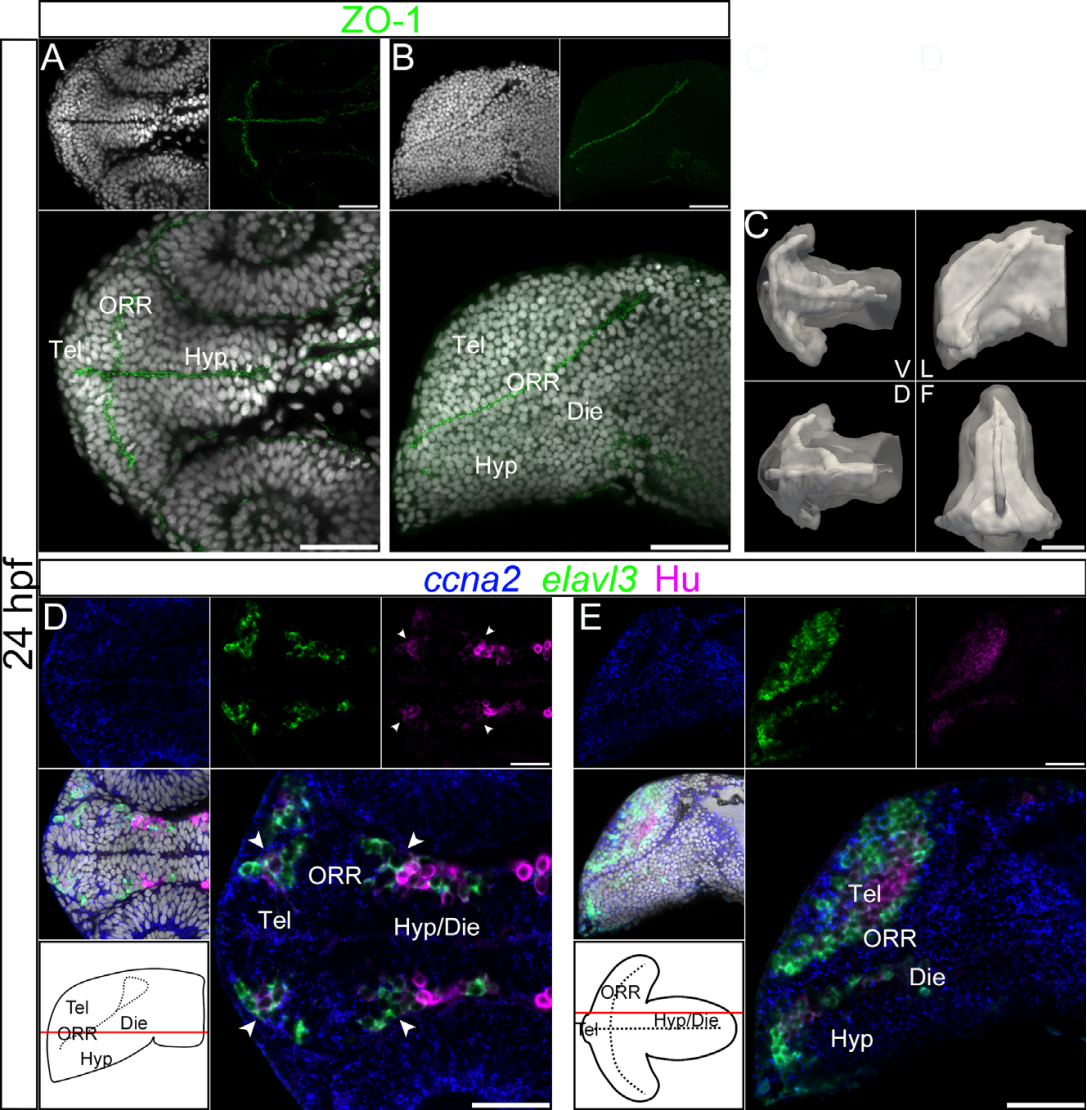
Ventricular organization and the centrifugal gradient of neurogenesis at 24 hpf. (A–B): Single confocal plane of a 24 hpf embryonic forebrain stained with DAPI (gray) and immunolabeled for ZO-1, in ventral (A) and lateral (B) views. diencephalon (Die); hypothalamus (Hyp); optic recess region (ORR); telencephalon (Tel). (C): Surface rendering of the shape of ventricle (white, in opacity), reconstructed from ZO-1 immunolabeling, overlapped on the shape of the brain (white, in transparency), reconstructed from DAPI staining. V = ventral, D = dorsal, L = lateral and F = frontal views. At 24 hpf the organization of the ventricular system is considerably simpler than at 48 hpf. The telencephalic ventricle rostral to the optic recess is not yet expanded (in contrast to 30 hpf). (D–E): Single confocal plane of a 24 hpf embryonic forebrain labeled for the neurogenic markers *ccna2*, *elavl3* and HuC/D, in ventral (D) and lateral (E) views. Bottom left drawings show the level of corresponding optical plane. The *ccna2* staining is widely distributed while *elavl3* and especially HuC/D expressions are restricted to small peripheral territories. The arrow heads in D indicate Hu-expressing cells located at the boundaries of telencephalon/ORR and ORR/hypothalamus. Scale bars = 50 μm.

At 24 hpf the hypothalamus and ORR are easily identifiable but the telencephalon is more difficult to delineate (Fig. 3A). The optic recess is already extended laterally, while the ventricle dorsal to the optic recess is even smaller than at 30 hpf (Fig. 3C frontal). This indicates that, over time, the ventral part of the optic recess tends to narrow, while the dorsal part widens suggesting that the dorsal and ventral optic recess territories undergo different morphogenetic processes.

At 24 hpf most of the cells in the brain are proliferating, as highlighted by the large *ccna2*-positive domains, nevertheless, dividing nuclei are still located exclusively along the ventricles (Fig. S3). Similar to at 30 hpf, Hu-expressing cells (Fig. 3D, arrow heads) are located at the borders of the three cell masses (Fig. 3D and E).

Interestingly, the first differentiated cells (HuC/D-positive cells, shown in magenta in Fig. 3D and E) appear in the telencephalon earlier than in other secondary prosencephalic domains. A second population of HuC/D-positive cells is detectable later in the ORR and only later in the hypothalamus (except for its most anterior part), implying heterochronic differentiation of progenitors within the secondary prosencephalon.

### Differentiated cells define the boundaries of the three morphogenetic entities

Since neural differentiation occurs in a centrifugal manner, from the ventricular wall to the mantle, we reasoned that if the telencephalon, the ORR and the hypothalamus are distinct morphogenetic entities, differentiated cells would be located and apposed at the border of each of the regions.

**Figure 4:**
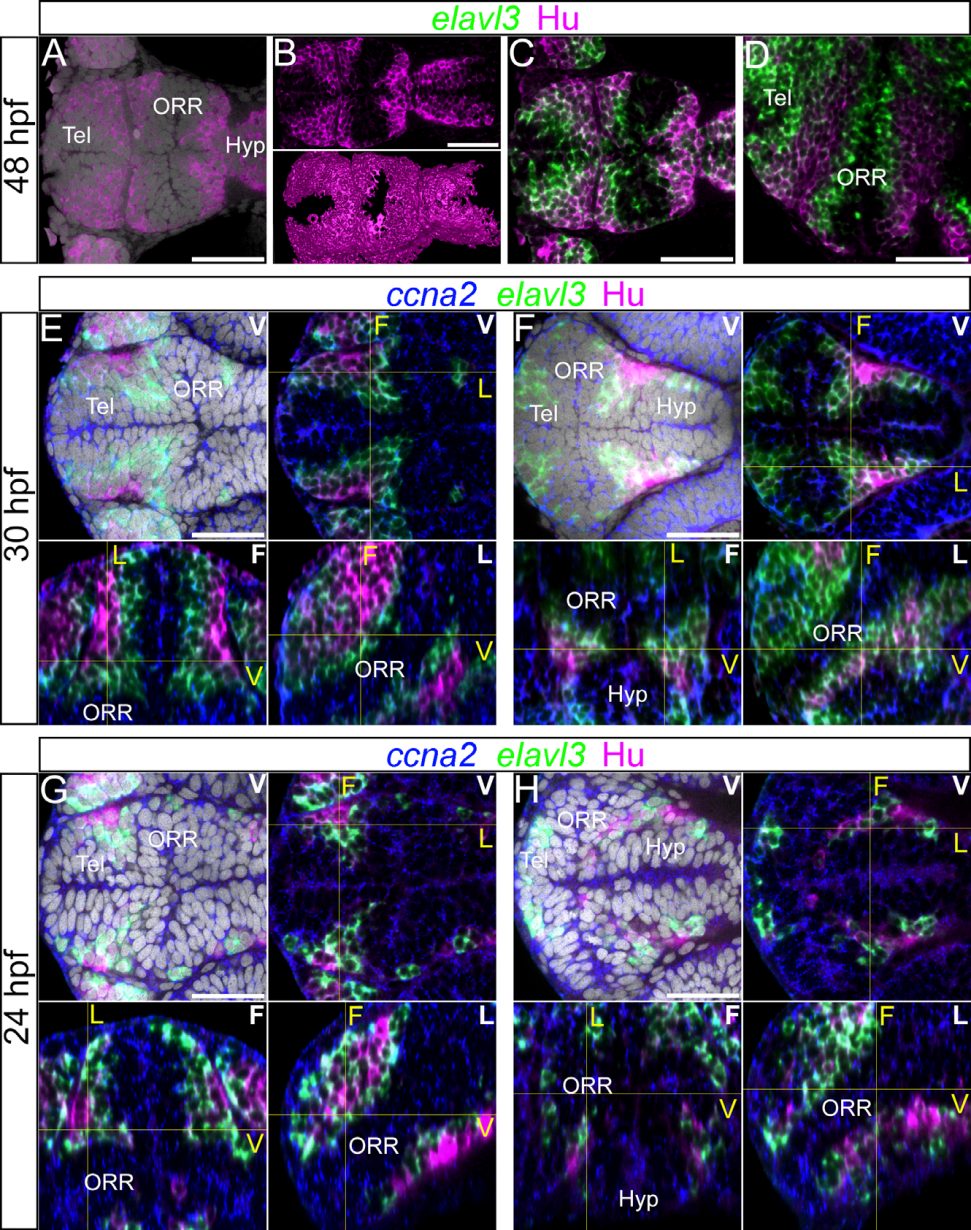
Differentiated cells mark the boundaries of each morphogenetic entity of the secondary prosencephalon. (A–D): 48 hpf forebrain labeled for *elavl3* and HuC/D superimposed on DAPI staining (gray). (A–B): HuC/D labeling with DAPI (A) and without DAPI (B). The top panel of B shows a single confocal plane of a ventral view and bottom panel of B shows the surface rendering of segmented HuC/D immunolabeling alone. These images show that the HuC/D staining itself is able to recapitulate the outline of the three morphogenetic entities. (C–D): Single confocal plane of a ventral (C) and lateral (D) view of *elavl3* and Hu labeling. Two rows of HuC/D-positive cells are in apposition at boundaries: the boundary between the telencephalon (Tel) and the optic recess region (ORR), and the boundary between ORR and the hypothalamus (Hyp). (E–H): Single confocal plane of the forebrain labeled with the neurogenic markers *ccna2*, *elavl3* and HuC/D at 30 hpf (E–F) or 24 hpf (G–H) in different views. In each case, the ventral view (indicated “V” in white) is shown on the top (with DAPI on the left, without DAPI on the right). The bottom images show the frontal (indicated “F” in white) and lateral (indicated “L” in white) views reconstructed using Z-projections of the ventral images. As they are ventral, frontal and lateral views from a single embryo, corresponding section levels of different views are indicated in yellow lines and yellow letters. As shown at 48 hpf (A–D), two layers of HuC/D labeling are detected around the boundary of the three cellular masses however this is not visible at the same section level. E and G show HuC/D and *elavl3* expression at the telencephalic/ORR border whereas F and H show at the ORR/hypothalamic region border. Scale bars = 50 μm.

At 48 hpf, the limit between each one of the three regions, the telencephalon, the ORR and the hypothalamus, is composed of at least two rows of Hu-expressing cells bordered on each side by *elavl3*-expressing cells (Fig. 4A–D). Thus, the caudal limit of the telencephalic differentiation domain meets the rostral edge of the ORR differentiation domain (they are orthogonally oriented), each of these domains being characterized by the presence of HuC/D-labeled cells. This is also the case at the limit where the caudal ORR differentiation domain abuts the rostral end of the hypothalamus differentiation domain.

The analysis at earlier stages further clarified how these borders are established over time. At 30 hpf, the rostral limit of the ORR with the telencephalon (Fig. 4E), as well as its caudal limit with the hypothalamus (Fig. 4F), is marked by the presence of HuC/D-positive cells sandwiched between two neurogenic domains. This is verified in all three dimensions, as seen on frontal and lateral reconstructed views (Fig. 4E and F, the three right and bottom panels). Similar characteristics were observed at 24 hpf (Fig. 4G and H), although at this stage, HuC/D-positive cells are mostly found as a single row of cells surrounded by rows of differentiating neurons.

The edge of the ORR, marked by the HuC/D-positive cells, is adjacent to the bundles of fibers immunolabeled for acetylated-tubulin, as shown in Fig. 1C. Therefore, the borders of the ORR with the telencephalon and the hypothalamus are marked in a highly congruent fashion by the morphology of the domains surrounding specific portion of the ventricle, the bundles of neuronal fibers, the presence of differentiated neurons, as well as the dense packing of radial glia processes as described in a different context^31^. Overall, these results confirm that the ventral secondary prosencephalon of the zebrafish brain displays three morphogenetic entities, each organized in a symmetrical and centrifugal manner around a part of the brain ventricles.

### Expression patterns of developmental genes in the secondary prosencephalon

We then examined how these three morphogenetic entities correlate with the expression pattern of genes widely used as regional markers in the prosencephalon.

**Figure 5:**
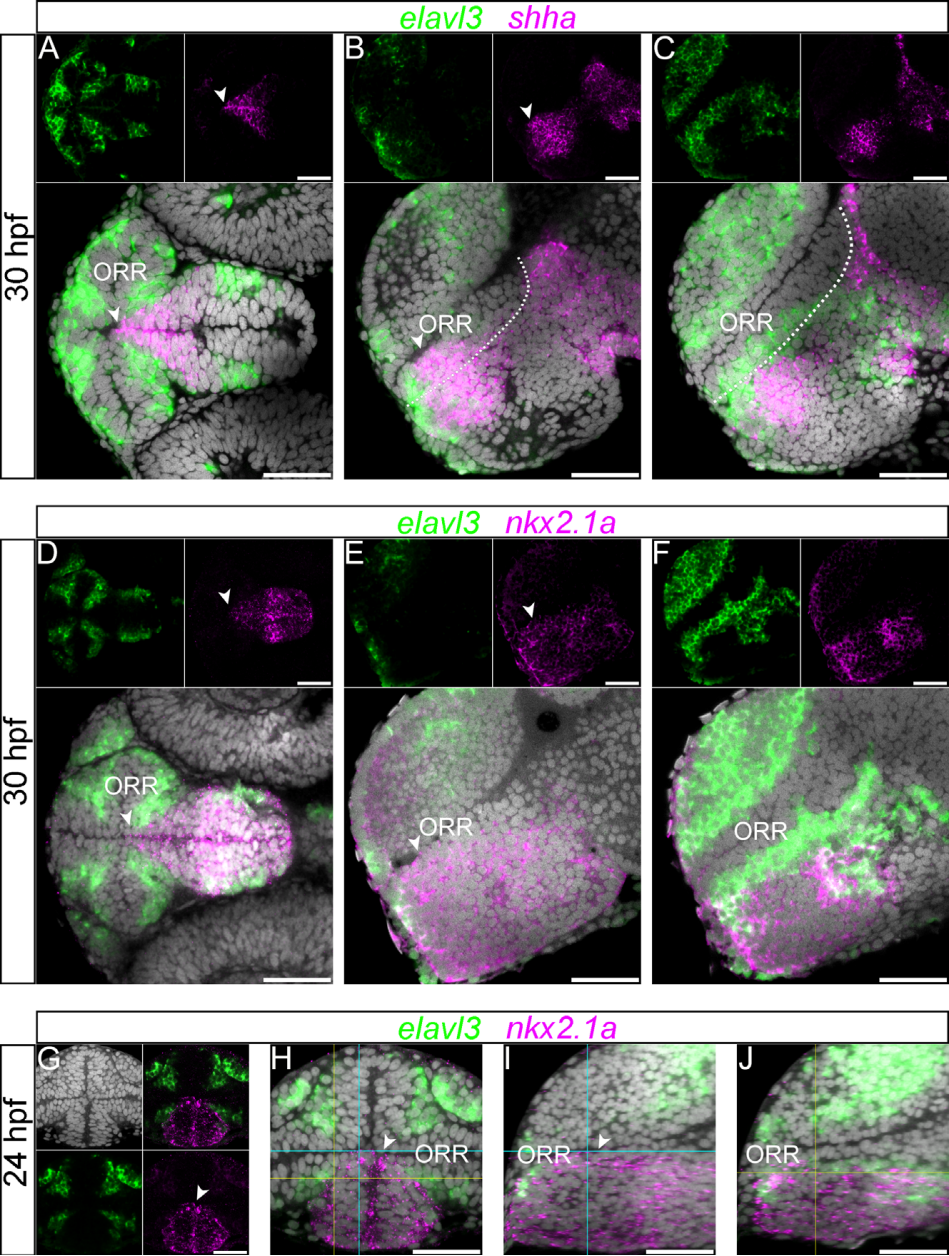
Expression of *nkx2.1a* and *shha* in the hypothalamic region. (A–C): 30 hpf forebrain following *elavl3* and *shha* *in situ* hybridization and DAPI staining (gray). The *shha* expression in the rostral edge of the hypothalamus displays a conical shape inserted into the ORR, which is visible in the ventral view (A). The expression reaches the ventricle at the most medial level (arrow heads of A and B), but not at a more lateral level (C).The currently proposed alar/basal limit is shown in dotted lines in the lateral sections (B and C), and it does not completely match the expression of *shha* in the anterior part of the forebrain. Scale bars = 50 μm. (D–F): 30 hpf forebrain following *elavl3* and *nkx2.1a* *in situ* hybridization and DAPI staining (gray). The expression domain is also conical shaped in the ventral view (D), and it reaches the ventricle at the most medial level (arrow heads of D and E), but not at a more lateral level (F). (G–J): 24 hpf forebrain following *elavl3* and *nkx2.1a* *in situ* hybridization and DAPI staining (gray) illustrated in a single confocal plane of a frontal view (G and H) and two lateral views (I and J) reconstructed using Z-projections of the frontal images. Corresponding section levels of the two lateral views are indicated in blue (more medial) and yellow (more lateral) lines. The expression domain of *nkx2.1a* has a conical shape, visible in the frontal view (G and H). Its dorsal limit reaches the ventricle only in the section close to the midline (arrow heads in G–I) and not in the more lateral section (J).

In all vertebrates studied so far, *Shh* is expressed all along the basal plate in the rhombencephalon and spinal cord. Since *Shh* expression extends into the forebrain, it has been proposed to also define a basal plate component in the secondary prosencephalon^24^,^32^,^33^. As previously described, *shha* is expressed within the anterodorsal hypothalamic region [7, 14, 22]. In frontal and ventral views, the expression domain of *shha* takes the conical shape of the rostral hypothalamus abutting the medio-caudal part of the ORR that surrounds it (Figs. 5A–C, S4A–B). As a consequence, the shape of the *shha*-positive hypothalamic region varies on sagittal sections according to their medio-lateral position: the *shha* expression domain reaches the optic recess on mid-sagittal sections (Fig. 5B, arrow head), but not on more lateral sections (Fig. 5C), where a large layer of interposed *elavl3*-positive and ventricular cells are positioned between the rostral end of the *shha* hypothalamic region and the optic recess. This expression domain does not change significantly from 24 hpf to 48 hpf (Fig. S4).

*Nkx2.1* codes for a transcription factor known to be downstream of Shh signaling, and it is expressed in a hypothalamic domain that partially overlaps with that of *Shh*^21^,^22^,^34^,^35^. In the rostral domain abutting the ORR, we found the expression of *nkx2.1a* is very similar to that of *shha* at all time points analyzed (Figs. 5D,G,H, and S4).

Interestingly, the expression domain of *shha* and *nkx2.1a* is consistently excluded from that of *elavl3*, most notably at the border between the ORR and hypothalamus (Fig. 5A,C,D,F). Thus, *shha* and *nkx2.1a* label most of the hypothalamic region, but they do not precisely mark the boundary between the ORR and the hypothalamus, since they are not detected in the last row of differentiating cells at the periphery of the hypothalamus when analyzed at a cellular resolution.

**Figure 6:**
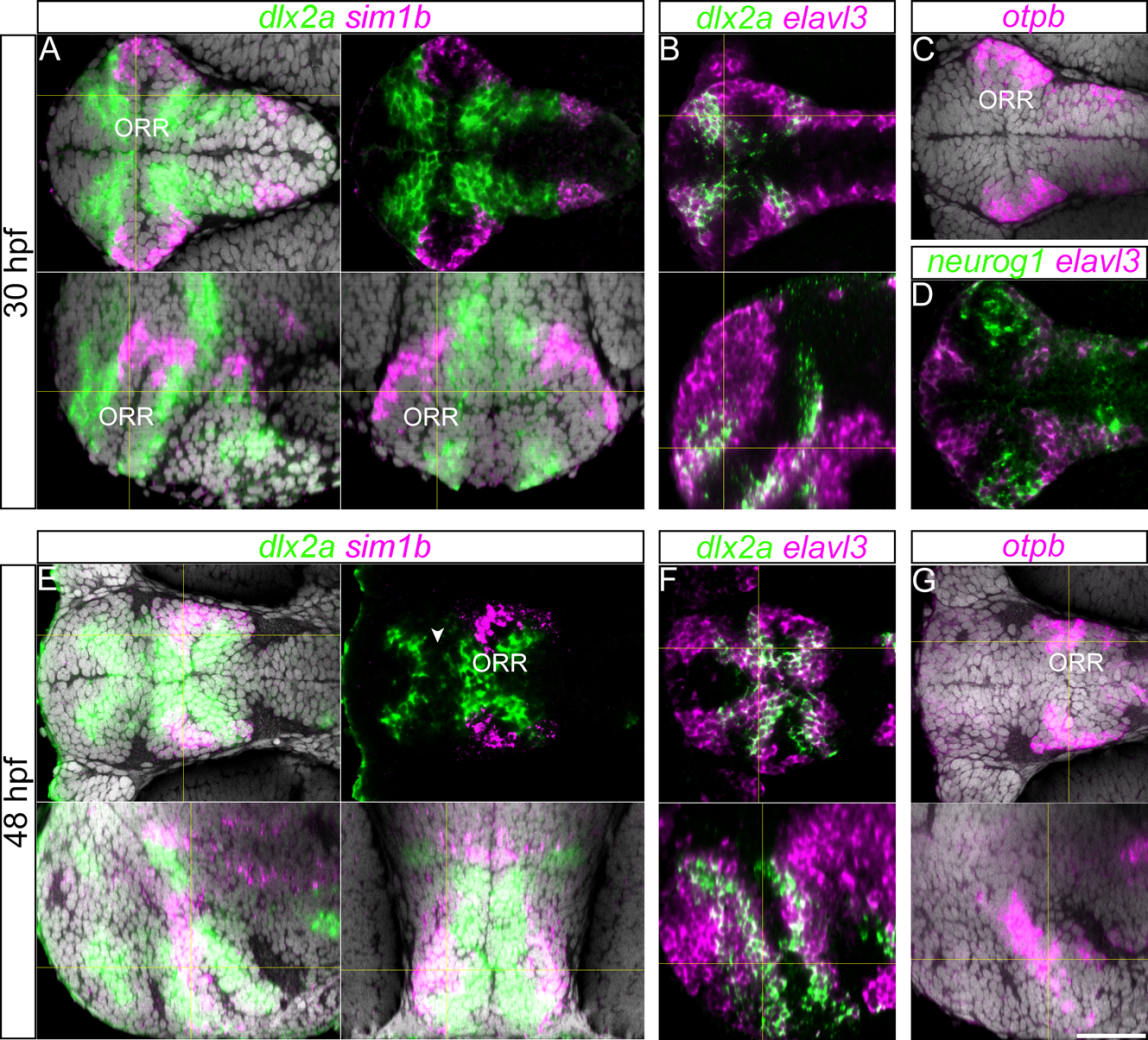
Gene expression along the ORR. (A): 30 hpf forebrain following *dlx2a* and *sim1a* *in situ* hybridization and DAPI staining (gray) on a single confocal plane of a ventral (top panels), lateral (left bottom panel) and frontal views (right bottom panel) reconstructed using Z-projections of the ventral images. Corresponding section levels are indicated in yellow lines (same for B, E–G). (B): Single confocal plane of a 30 hpf forebrain following *dlx2a* and *elavl3* *in situ* hybridization in ventral view (top panel) and lateral view reconstructed using Z-projections of the ventral images (bottom panel). (C): Single confocal plane of a 30 hpf forebrain following *otpb* *in situ* hybridization and DAPI staining (gray) illustrated in a ventral view. In the ORR, the expression pattern of *otpb* is similar to the *sim1a* staining. (D): Single confocal plane of a 30 hpf forebrain following *neurog1* and *elavl3* *in situ* hybridization in ventral view. In the ORR, the expression pattern of *neurog1* is similar to the *sim1a* and *otpb* staining covering the lateral end of the region. (E): 48 hpf forebrain following *dlx2a* and *sim1a* *in situ* hybridization and DAPI staining (gray), single confocal plane of ventral view (top panels) and of lateral (left bottom panel) and frontal (right bottom panel) views reconstructed using Z-projections of the ventral images. The arrow head in the right top panel indicates the gap of two *dlx2a*-expressing domains in the telencephalon and ORR. (F): 48 hpf forebrain following *dlx2a* and *elavl3* *in situ* hybridization in a single confocal plane of ventral view (top panel) and of lateral view reconstructed using Z-projections of the ventral images (bottom panel). (G): 48 hpf forebrain following *otpb* *in situ* hybridization and DAPI staining (gray) on a single confocal plane of ventral view (top panels) and of lateral view reconstructed using Z-projections of the ventral images (bottom panel). Corresponding section levels are indicated in yellow lines.

We next analyzed the expression of *Sim1*, *Otp* and *Dlx2* genes as they have been shown to be expressed within subdivisions of the so-called alar hypothalamus in several groups of vertebrates^8^,^22^, and *Dlx2* is also a marker of the subpallium within the telencephalon. In zebrafish embryos at 30 and 48 hpf, we found that the *dlx2a* expression domain overlaps with the *elavl3*-expressing domains at the border of the three morphogenetic entities (Fig. 6B and F). Additionally, at 48 hpf the rostral *dlx2a* expression domain in the telencephalon is entirely distinct from the *dlx2a* domain in the ORR (Fig. 6E, arrow head indicating the gap). These data suggest that the *dlx2a*-positive domains are composed of differentiating cells originating from the ventricular zones of the three distinct morphogenetic entities.

*Sim1a* and *otpb* appear to be expressed in the same region around the lateral edge of the optic recess (Fig. 6A and C). Their expression patterns are very dynamic during development (Fig. S5), however, at 30 hpf and 48 hpf, *sim1a* expression is consistently in apposition to *dlx2a* expression (Fig. 6A and E). The dynamics of *dlx2a* expression can be determined by comparing the ventral views of 30 hpf (Fig. 6A and B) and 48 hpf (Fig. 6E and F). In the ORR, *sim1a* and *otpb* expression is located within the *elavl3*-expressing domain (Fig. 6) and largely coincides with the expression of the proneural gene *neurogenin1* (*neurog1,* Fig. 6D) suggesting that they are predominantly expressed in differentiating cells. On the lateral sections presented in fig. 6, the *sim1a* expression domain is flanked by two *dlx2a* domains, whereas in the ventral and frontal sections *sim1a* is located at the lateral edge of the *dlx2a* domain. Together, the expression pattern of these developmental genes further supports the ORR being a distinct morphogenetic entity.

**Figure 7:**
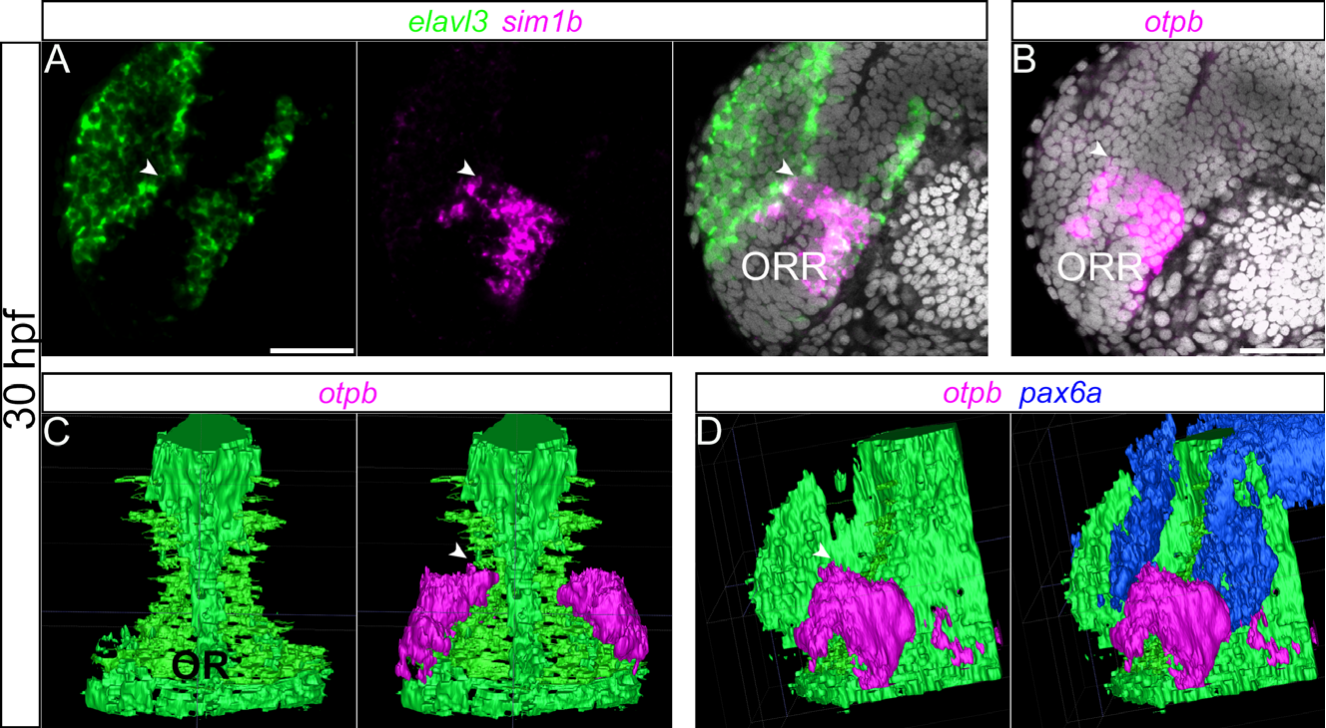
3D demonstration of the subdivision in the ORR. (A–B): Single confocal plane of a lateral view of a 30 hpf forebrain following *elavl3* and *sim1a* (A) or *otpb* (B) *in situ* hybridization and DAPI staining (gray). (C–D): 3D rendering from confocal images following *otpb* and *pax6a* *in situ* hybridization and DAPI staining, illustrated in a frontal (C) and a lateral (D) views. Ventricle shape of the optic recess (OR) was deduced from DAPI staining and segmented manually using ITK-SNAP 2.4.0. *otpb*- and *pax6a*-positive domains were segmented semi-automatically using ITK- SNAP 2.4.0. The expression of *sim1a* and *otpb* delineates the dorsal limit of the ORR (arrow heads), which fits the inversion of the curvature of the ventricle (C and D). *Pax6a* (D), which is known to be expressed in the dorsal domain of the forebrain, shows the complementary expression pattern with *otpb*. Scale bars = 50 μm.

The 3D reconstruction of the optic recess morphology combined with that of *sim1a*/*otpb* gene expressions (Fig. 7A and B) demonstrates that their dorsal limit coincides with the dorsal limit of the optic recess, marked by the inflection point in the curvature of the ventricle (Fig. 7C, arrow head). We also found that the expression of *pax6a*, a well-known dorsal marker in the forebrain, is abutting the dorsal limit of the ORR (Fig. 7D). Together this data strengthens the regional identity of the ORR and sets its dorsal limit.

**Figure 8:**
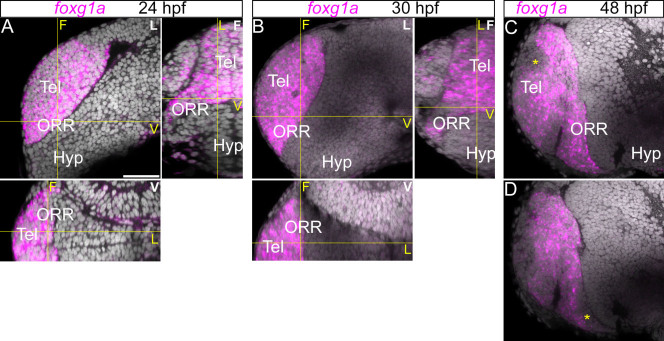
Expression of *foxg1a* in the telencephalic region. Anterior forebrain region following *foxg1a* *in situ* hybridization and DAPI staining (gray) at 24 (A), 30 (B) and 48 hpf (C). (A–B): A single confocal plane of a lateral view (left top panel; indicated “L” in white) and the frontal (right top; indicated “F” in white) and ventral (bottom panel; indicated “V” in white) views reconstructed using Z-projections of the lateral images. Corresponding section levels are indicated in yellow lines and yellow letters. The *foxg1a* is expressed in the entire region antero-dorsal to the optic recess. (C–D): A single confocal plane of two different lateral views at 48 hpf (C more medial and D more lateral). The expression of *foxg1a* is reduced at 48 hpf in some territories (*). Scale bar = 50 μm.

**Figure 9:**
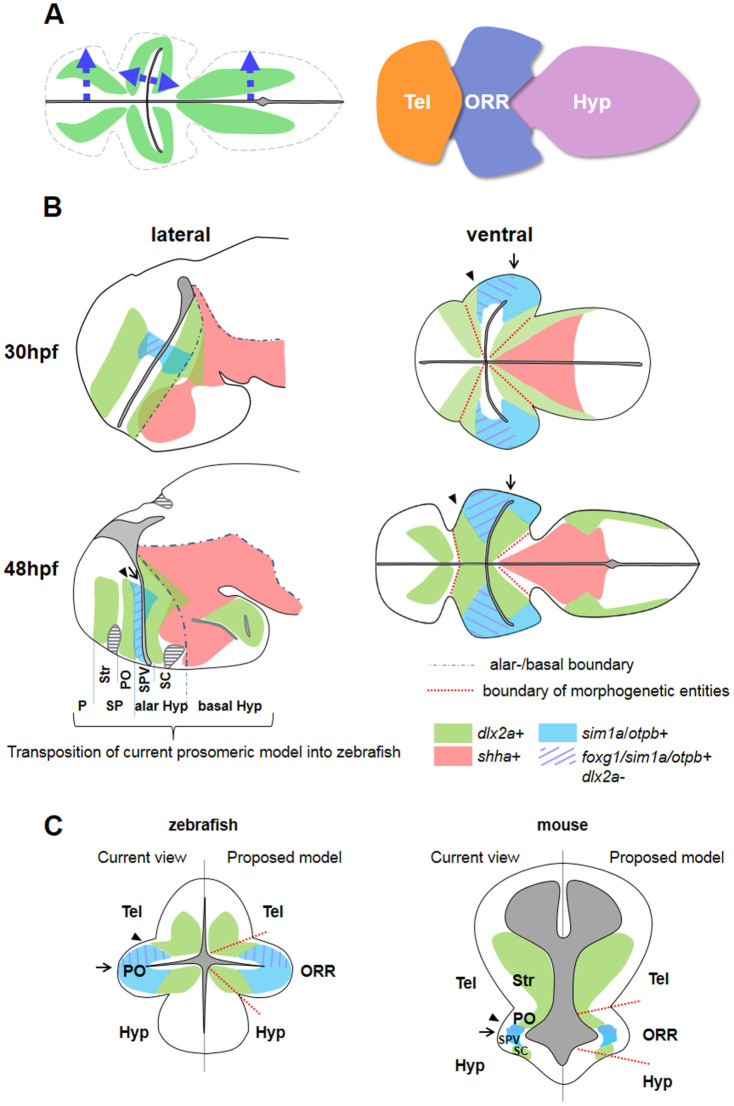
Schematic drawing summarizing the results of this study. (A): Proposed model of basal forebrain regionalization. The morphogenesis follows the ventricular organization, forming three masses of cells differentiating (green) in the direction of arrows. The secondary prosencephalon thus contains three morphogenetic entities, the telencephalon (Tel), the optic recess region (ORR), and the hypothalamus (Hyp). (B): The expression patterns of several genes examined in this study at 30 hpf and 48 hpf zebrafish embryos, on lateral (left) and ventral (right) views. The black dots in the lateral views represent the alar/basal boundary proposed in the current prosomeric model. The red dotted lines in the ventral view indicate the boundaries of the morphogenetic entities in this study. Annotation on the lateral view at 48 hpf is a transposition from the proposed prosomeric model defined in tetrapods. However, such longitudinal segmentation is not recognizable from the ventral view. Comparison between 30 and 48 hpf also indicates that the regional identity delineated by genes (e.g. SPV by *sim1*/*otp* expression) is only transient. (C): Schematic frontal view of zebrafish and mouse forebrain. The gene expression data in mouse is based on previous publications^6^,^41^,^46^,^47^,^66^. In the current view (left side) the secondary prosencephalon is divided into the telencephalon and the hypothalamus. However there is a discrepancy in depicting the boundary using developmental genes. The arrow heads indicate the boundary based on *Dlx* and *Sim*/*Otp* expression territories, whereas the arrows indicate the boundary based on *Foxg1*. Identification of the “preoptic area” (PO) in teleosts and tetrapods are also different. In the proposed model (right side) based on our zebrafish study, the regional boundaries are depicted by apposed differentiated cells originating from different ventricular zones, instead of the gene expression only. Representation of arrows, arrow heads, and the color code for genes are the same in B and C. Abbreviations: Hyp: hypothalamus, ORR: optic recess region, P: pallium, PO: preoptic area, SC: suprachiasmatic area, SPV: supraopto-paraventricular area, SP: subpallium, Str: striatum, Tel: telencephalon.

*Foxg1* is often used as a marker to identify the telencephalon in non-mammalian species including zebrafish^36^,^37^. This is due to the prominent expression of *Foxg1* in the telencephalic hemispheres in mouse^38^,^39^, however, its expression has been observed to extend to the nasal (anterior) part of the optic stalk and of the optic vesicle^40^,^41^. In line with these observations, we found that the *foxg1a* is expressed in the entire region rostrodorsal to the optic recess, containing the telencephalon and the rostral part of the ORR (Fig. 8). Its caudoventral limit does not coincide with that of *dlx2a*, a subpallial marker in the telencephalon, and overlaps with *sim1a*/*otpb*, alar hypothalamus markers (Fig. 9).

Overall, these data show that, as opposed to relying on a single marker, analyzing the intersecting and overlapping expression of a combination of genetic markers allows for the most accurate definition of the regional boundaries in the secondary prosencephalon.

## Discussion

To be able to fully account for the highly dynamic changes taking place during regionalization of the zebrafish prosencephalon, we chose to analyze the brain morphology in relation to the ventricular organization, with cell state information and gene expression patterns. The 3D reconstruction and segmentation of the secondary prosencephalon in zebrafish revealed the unexpected complexity of the ventricular morphology, in particular that of the optic recess. The morphogenetic organization of the secondary prosencephalon is thus very difficult to interpret without 3D analysis of the data at cellular resolution. The present study revealed the existence of a morphogenetic entity surrounding the optic recess, the optic recess region (ORR), distinct from the telencephalon and the hypothalamus (Fig. 9A).

A “morphogenetic entity” can be defined as a natural unit, built from a coherent neurogenic process, with the formation of clear borders with neighboring areas. Its spatial organization directly derives from the ventricle-to-mantle orientation of the proliferation and differentiation stages of neural progenitors over time. The borders of such a morphogenetic entity are marked by 1) bundles of fibers, 2) concentration of radial glia processes and 3) apposition of differentiated neurons coming from different “morphogenetic entities”, as it is the case for the telencephalon, ORR and hypothalamus, respectively. Accordingly, such a morphogenetic entity would also express a characteristic set of genes, which underlie the neurogenic, morphogenetic and differentiation processes.

The observations we report here that the zebrafish preoptic area is a morphogenetic entity located between the telencephalon and the hypothalamus fit with classical anatomical descriptions of the adult teleost brain. This region has been identified as the “optic stalk” in zebrafish embryonic brains by Wilson *et al.*^4^,^42^. In agreement with our observation, these authors show that a gap exists between the telencephalon, identified by *foxg1a* expression (see below), and the hypothalamic boundary depicted by the conical-shape of *shha* expression. However, this gap domain was originally not considered to be a region in itself. According to these observations, the ORR could derive from the eye field of the neural plate. Studies performed at earlier developmental stages indeed indicate that the optic vesicles originate from the same topological position as the ORR, between the telencephalon and the hypothalamus^43^,^44^,^45^.

We propose that, during brain patterning, differentiated cells arising from different morphogenetic entities abut to contribute to the formation of the regional boundaries between telencephalon, ORR and hypothalamus. In this respect, the formation of these regions does not preclude the possibility that cells could migrate across the boundaries between the telencephalon, ORR and hypothalamus, but such migration occurs at later developmental stages than those we studied here. It should be stressed that the regional boundaries cannot always be delineated just based on gene expressions. We found that *foxg1a* is expressed in the telencephalon and the rostral ORR. Around 30 hpf, *sim1a* and *otpb* are prominently expressed in the ORR, but they are not detected in the differentiating (*elavl3*-positive) cells at the border of ORR with the telencephalon and with the hypothalamus. Similarly, *nkx2.1a* is expressed in the hypothalamic region, but not present in the differentiating cells at the border with the ORR.

Interestingly, the differentiating cells at the borders of the telencephalon/ORR and the hypothalamus/ORR both express *dlx2a*. At these developmental stages, *dlx2a* expression is found exclusively in the *elavl3*-positive domains. Based on the spatial orientation of cell maturation, we interpret that *dlx2a*-expressing cells in the telencephalon, ORR, and hypothalamus represent differentiating cell populations that originate from the ventricular zones of the three distinct morphogenetic entities. Although *dlx2a* is often used as a regional marker, it is likely that it reflects stage of cell differentiation, in addition to positional identity.

Our combined analysis of cell differentiation markers and patterning gene expression, conducted at cellular resolution, leads to a reassessment of the interpretation of the neural genoarchitecture^18^ to identify homologous forebrain regions among different vertebrate classes. According to the current prosomeric model, the rostral *Dlx2* expression domain is identified as the subpallium (containing the striatum and preoptic area), the *Sim1*/*Otp*-expressing domain as the supraopto-paraventricular area (SPV), and the caudal *Dlx2* domain as the suprachiasmatic area (SC). Although not fully fitting with the limit of *Shh* expression, the alar/basal boundary is usually drawn in direct continuity from the rhombencephalon all the way to the terminal wall of the neural tube. Within this framework, the SPV and SC together have been considered as the alar hypothalamus, and the *Shh*/*Nkx2.1* domains ventral to the SC as the basal hypothalamus.

Previous work suggested that the expression patterns of developmental genes in zebrafish are comparable with those in tetrapods, when lateral views and/or sagittal sections were used^8^,^23^,^24^,^46^,^47^. However, our analysis reveals discrepancies in applying the current model of forebrain regionalization to depict regional boundaries in zebrafish. Firstly, the *sim1a*/*otpb*-expressing domain, flanked by *dlx2a*-expressing domains (used to define the SPV) is recognizable only transiently (at 48 hpf) from a lateral view (Fig. 9B). Secondly, the “preoptic area” (PO) defined in zebrafish atlases^25^,^28^ is much larger than the PO transposed from the current prosomeric model. Whereas the tetrapod PO is limited to the *Dlx2*/*Foxg1* positive domain and considered to be a part of the telencephalon (Fig. 9C, Current view in the mouse brain), the teleost PO includes the *sim1a/otpb* domain (Fig. 9C, Current view in the zebrafish brain). A recent publication in zebrafish suggests that *otp*-expressing neuroendocrine cells in the PO are homologous to those located in the alar hypothalamic SPV in tetrapods^27^, instead of being telencephalic. Thirdly, there is a discordance in the telencephalic/hypothalamic boundary when it is defined based on gene expressions. The telencephalic limits depicted by *dlx2a* (Fig. 9B and C, arrow heads) and by *foxg1a* (Fig. 9B and C, arrows), the two commonly used marker genes, do not coincide. In agreement with our observation, such a difference in the ventral limit of the *Dlx2* and *Foxg1* expression territories, although small, has already been described in the mouse brain^6^,^41^ (Fig. 9C, mouse).

Considering the ORR as a morphogenetic entity distinct from the telencephalon or the hypothalamus would resolve such discrepancies in the definition of regional identities. Our model of anterior forebrain regionalization in comparison with the current model is shown in Fig. 9C. Our hypothesis considering a subset of the tetrapod “alar hypothalamus” as a part of the ORR is consistent with the previous study suggesting that the *otp*-positive neuroendocrine cells in tetrapods and teleosts are homologous^27^. The *dlx2a*-positive/*otpb*-negative area of the anterior ORR may be homologous to the non-evaginated “telencephalic stalk area” in amniotes^48^,^49^. Importantly, this new model of brain regionalization relies on a radially-organized morphogenesis centered by and organized around the ventricles. This radial organization would have been difficult to recognize in tetrapods due to the relative small size of the ORR compared to the enlarged telencephalon.

Careful observations of published data, nonetheless, indicate the presence of the ORR in tetrapods. An area flanked by the anterior commissure and the postoptic commissure (here identified as the ORR) is present in mouse developing brains, and it has been called the “optic stalk”^33^. The rostral half of this region expresses *Foxg1*^33^,^40^,^41^, similarly to the rostral ORR in zebrafish. The presence of the ORR in tetrapods needs to be assessed by carefully probing the morphogenesis occurring around the optic recess and lineage tracing in different groups of species.

Overall, the protocols we designed here for staining and imaging the forebrain in the zebrafish provide an unparalleled refinement in morphological and gene expression analyses at cellular resolution. A systematic application of these procedures will allow building a 3D atlas of the zebrafish forebrain at different time points during development, providing a powerful and comprehensive tool to analyze in detail morphogenesis, neurogenesis, and regionalization in the zebrafish brain, in a comparative perspective.

## Methods

### Fish strains

Wild-type zebrafish (AB strain) were raised according to standards procedures^50^. Embryos were staged as hours post fertilization (hpf) according to specific criteria outlined by Ref. 51. All experiments were carried out in accordance with animal care guidelines provided by the French ethical committee. The experiments were approved by the local Ethical Committee (n° 59) and carried under the supervision of authorized investigators (level 1 for experiments on model animals).

### Antibodies

Mouse anti-ZO-1 (clone ZO1-1A12, Invitrogen) was used at a dilution of 1/200 to label the ventricles^29^. Mouse anti-acetylated Tubulin (clone 6-11B-1, Sigma) was used at a dilution of 1/1000 to label axons^52^. Mouse anti-HuC/D (clone16A11, Invitrogen) was used at a dilution of 1/500 to label neurons^53^. Rabbit anti-GFAP (Z0334, Dako) was used at a dilution of 1/1000 to label glial processes.

### Whole-mount *Fluorescent in situ* *hybridization* and immunofluorescence

Fluorescent in situ hybridization (FISH) was performed as described previously^54^. Embryos were fixed in fresh 4% paraformaldehyde (PFA) in PBS-tween20 0.1% (PBST) for 24 hours at 4°C, dehydrated and stored at −20°C in methanol for at least 24 hours. Embryos were then rehydrated through a descending series of ethanol solutions, permeabilized by proteinase K (10 μg/ml; P6556, Sigma) treatment followed by incubation with 20 mM glycine in PBST. Embryos were prehybridized in hybridization buffer for 4 hours at 65°C. Hybridization was then performed at 65°C for 18 hours in hybridization buffer containing the mixture of probes. Samples were washed (50% formamide/50% 2xSSC; 2xSSC; 0.2xSSC; PBST), treated for 30 minutes with H_2_O_2_ 3% to inactivate endogenous peroxidases, and washed again in PBST. Probes detection was carried out as follow: 1) incubation with hapten-specific antibodies conjugated to POD, 2) incubation in H2O2 0.001% with the suitable fluorophore-conjugated tyramide, and 3) POD inactivation, by H_2_O_2_ 2%. Fluorescein-labeled probes were recognized by an anti-fluorescein POD antibody (11207733910, Roche Diagnostics) and revealed by a fluorescein-conjugated tyramide (protocol available on Xenbase: http://www.xenbase.org/other/static/methods/FISH.jsp). For digoxigenin- labeled probes, an anti-digoxigenin-POD antibody (11207733910, Roche Diagnostics), and a TAMRA-conjugated tyramide^55^ were used.

Antisense RNA probes for *sim1a*^56^ and *otp1b*^57^ were generated from RT-PCR products and cloned into pCR2.1 vectors (Invitrogen). The primer paires used for PCR are the following: 5′-GCA GCG GGT ACC TGA AGA T-3 and 5′-CGG AGA GAG TCT TGT TTT GGT C-3′ for *sim1a*, and 5′-GTA GAG TAG TTT GGG AAG CAG TTG TGA C-3′ and 5′-TTG GTT TTG CTG GCC GCC CGT CTG-′3 for *otpb*. Other antisense probes have been described in the following publications:*ccna2*^58^, *elavl3*^58^, *nkx2.1a*^34^, *shha*^59^, *dlx2a*^60^, *neurog1*^61^, *pax6a*^62^, and *foxg1a*^63^.

Immunofluorescence was performed after FISH: embryos were incubated in primary antibody in DMSO 0,5%,Triton 0,1%, goat serum 4%/PBST for 24 hours at 4°C. After washes, embryos were incubated in secondary antibodies for 4 hours at RT.

### Image acquisition

After extensive washes, embryos were counterstained with dapi (5 μg/ml, Sigma) mounted in Vectashield H-1000 Mounting Medium (Vector, Eurobio/Abcys) and examined using a Zeiss LSM700 laser scanning confocal microscope equipped with a *40 ×* 1.3 NA oil objective. Multitracking sequential acquisitions were performed to avoid signal crossover. Stacks ranged from 100 μm to 170 μm in Z-dimension with a step of 1 μm. Linear laser or/and PMT correction was used to compensate for signal attenuation in Z. Pinhole settings chosen to 1.0 Airy unit.

### Image processing

Images were first visualized using Fiji^64^ and then processed through ITK-SNAP 2.4.0^65^ software to perform manual and semi-automatic 3D segmentation of brain and ventricle, respectively. Reconstructed structures were then exported as binary 3D images and further processed with custom developed software to extract surfaces suitable to be embedded in a 3D pdf file. Images of ventricle underwent a median filtering and a morphological closure. All images were subsampled in order to simultaneously smooth and reduce the size of the resulting isosurfaces. Each image has been converted to a 3D pdf format through the 3Dpdf Paraview plugin (http://www.pdf3d.com/) and the final embedding in the 3D pdf file was performed via Adobe Acrobat X Pro utilities. The morphological closure facilitates the visualization of ventricles, but produces structures thicker than the corresponding ones in the raw data. We considered this compromise acceptable for our purpose of 3D interactive visualization.

## Supplementary Material

Supplementary Information supplementary dataset

Supplementary Information Interactive PDF file demonstrating ventricular morphology
