# Supplementary material for: Identification of the optic recess region as a morphogenetic entity in the zebrafish forebrain
Source: Sci Rep. 2015 Mar 4;5:8738. doi: 10.1038/srep08738 (PMC5390081; doi:10.1038/srep08738)
Supplement: Supplementary Information — supplementary dataset [file srep08738-s1.doc]

Identification of the optic recess region as a morphogenetic entity in the zebrafish forebrain

Pierre Affaticati^1^*, Kei Yamamoto^1^*, Barbara Rizzi^2^, Charlotte Bureau^1^, Nadine

Peyriéras^2^, Catherine Pasqualini^1^, Michaël Demarque1#, and Philippe Vernier1#

Unit Neurobiology & Development (UPR3294), Institute of Neurobiology Alfred Fessard, CNRS; Avenue de la Terrasse, 91190 Gif-sur-Yvette, France

Supplementary Figures S1 to S5


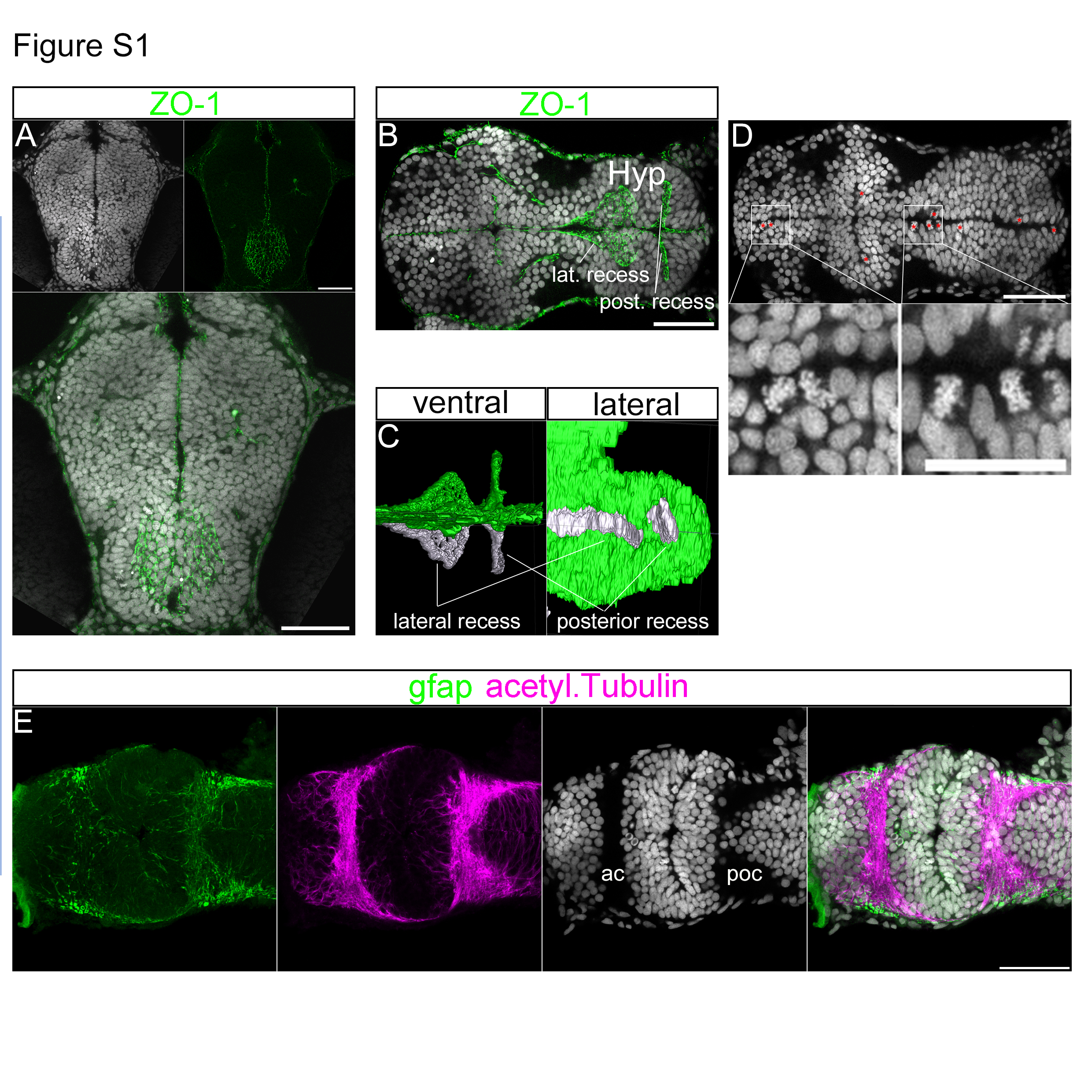


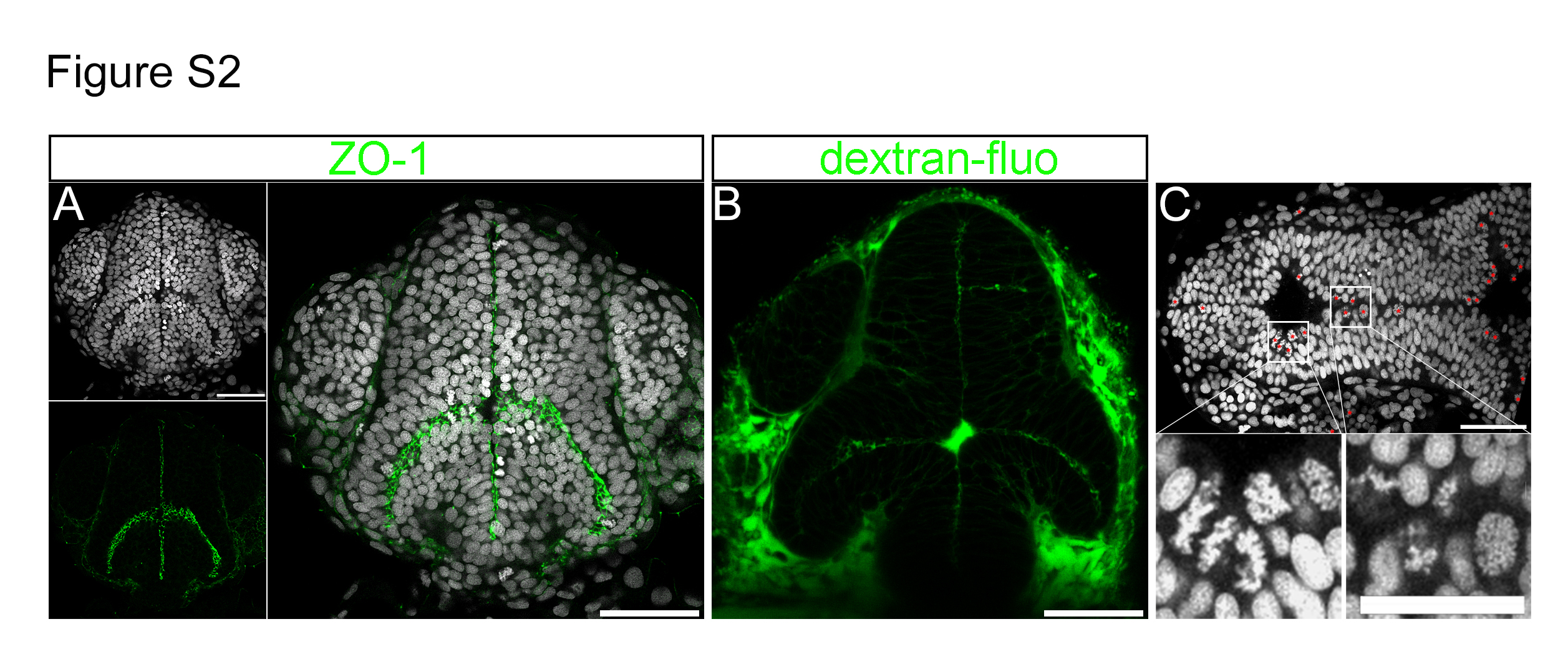


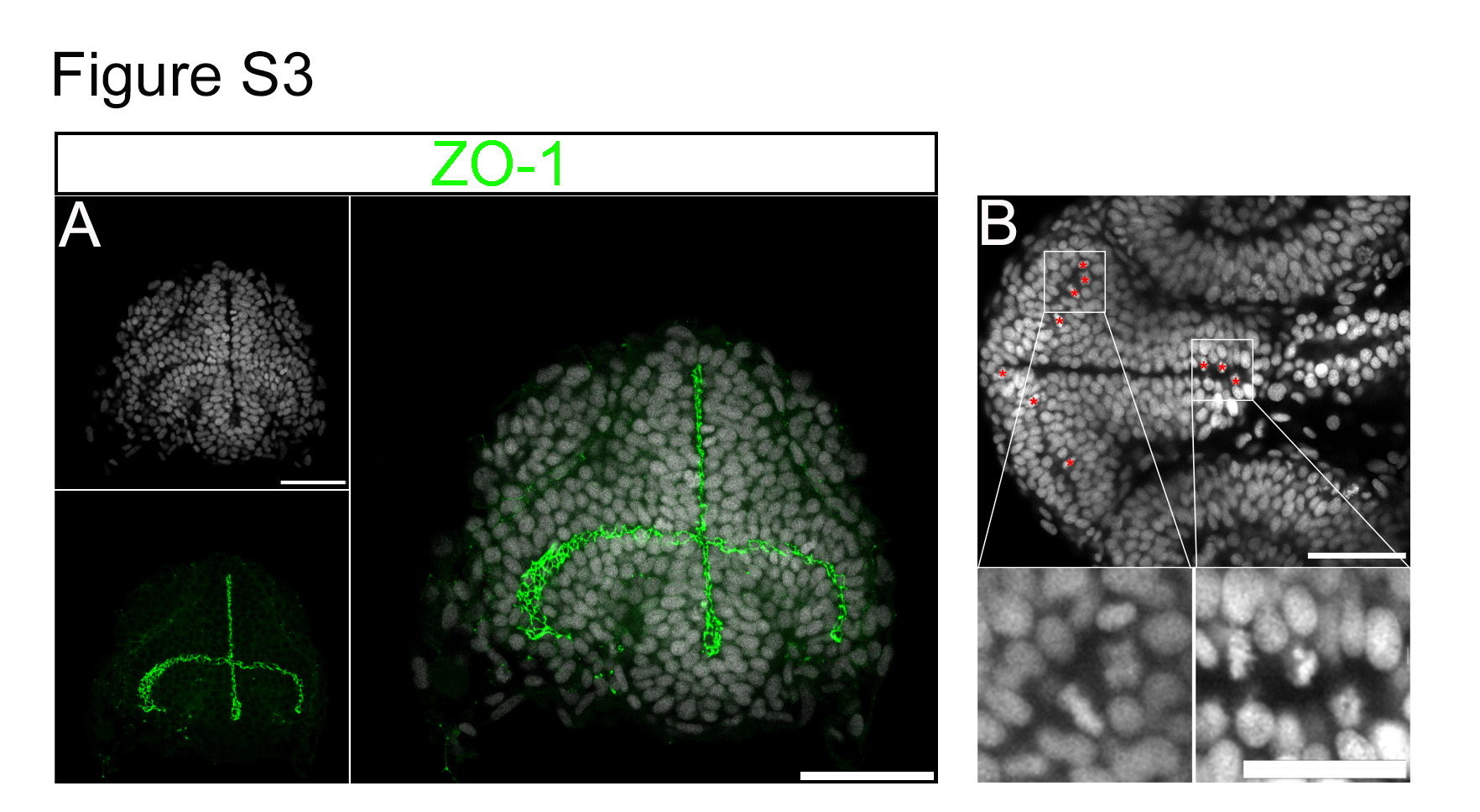


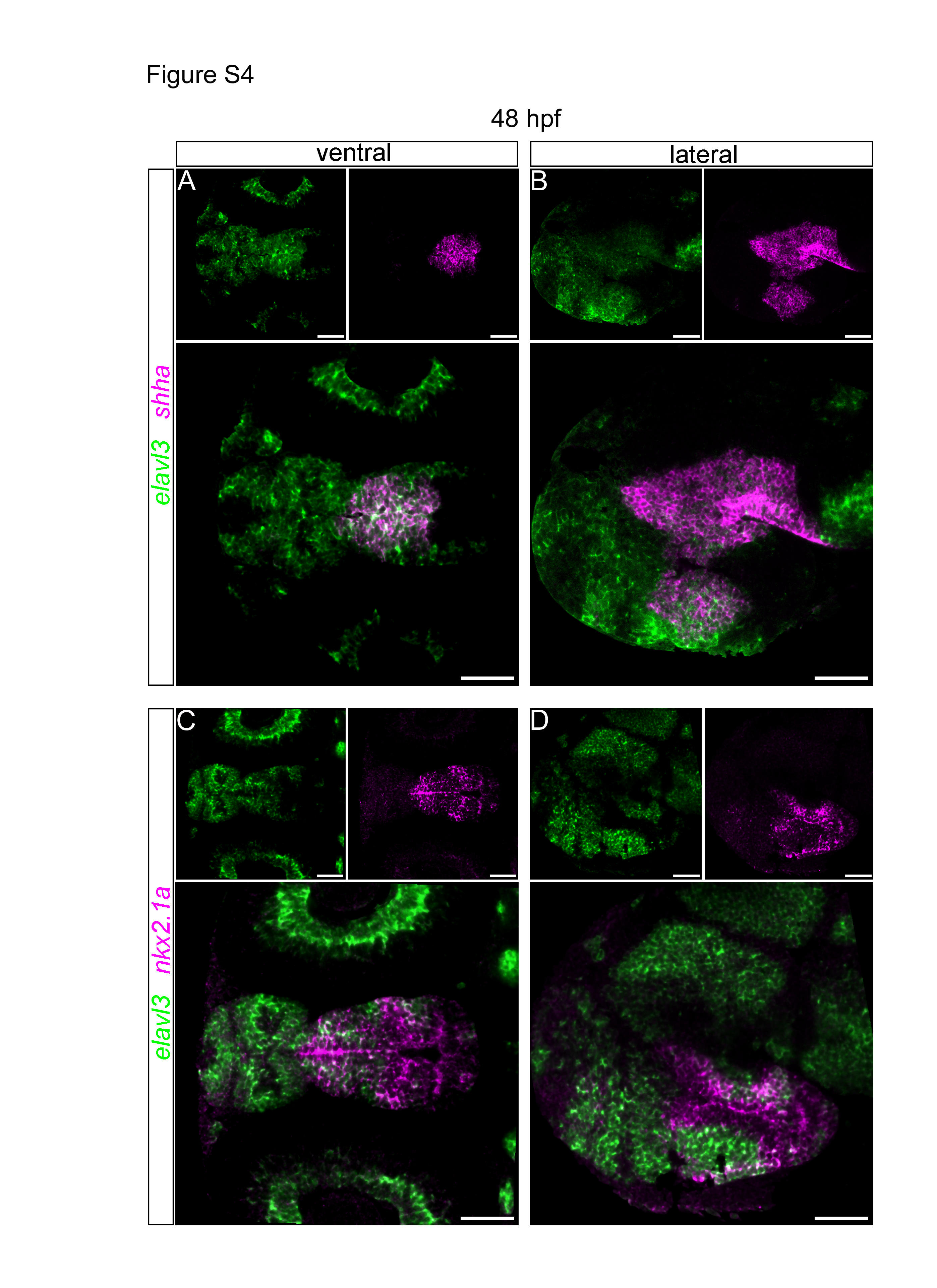


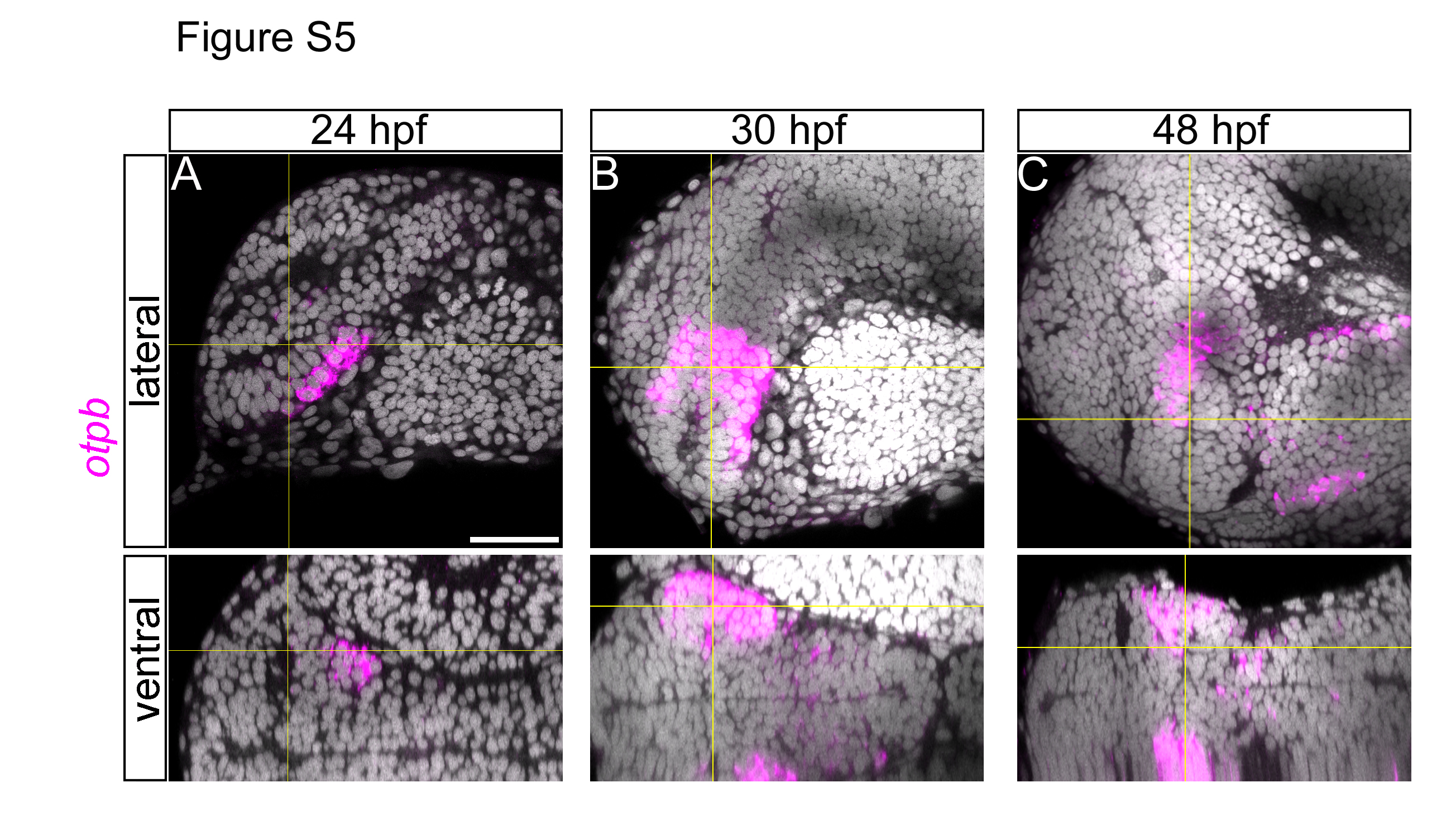


Legends of supplementary figures :

**Figure S1. Lateral and posterior recesses and mitosis taking place along the ventricle at 48 hpf.**

**A-B:** Single confocal plane of a 48 hpf embryonic forebrain displaying DAPI staining (gray) and immunolabeling for ZO-1. Frontal (A) and ventral (B) views at the level of lateral and posterior recesses. **C:** 3D semi-automatic segmentation of the ZO-1 immunolabeling in the lateral and posterior recesses using ITK- SNAP 2.4.0 software (Snake ROI tool). The right hemisphere and the midline are visualized in green, while the left hemisphere is visualized in white, to highlight the convoluted ventricular organization. **D:** Single confocal plane of a 48 hpf embryonic forebrain labeled with DAPI (gray). Top panel: red asterisks indicate cells dividing along the ventricle. Lower panels: higher magnification of the same cells. Scale bars = 50 µm.

**Figure S2. The optic recess and mitosis taking place along the ventricle at 30 hpf.**

**A:** Frontal view of a single confocal plane of a 30 hpf embryonic prosencephalon showing DAPI staining (gray) and immunolabeling for ZO-1. **B:** Frontal view of a single confocal plane of a live embryo (30 hpf), the ventricle of which has been filled-in with dextran-fluo. The image of the non-fixed embryonic brain confirms that the ventricular organization is not significantly modified after paraformaldehyde fixation. **C:** Single confocal plane of a 30 hpf embryonic forebrain stained with DAPI (gray). Top panel: red asterisks indicate cells dividing along the ventricle. Lower panels: higher magnification of the same cells. Scale bars = 50 µm.

**Figure S3. The optic recess and mitosis taking place along the ventricle at 24 hpf.**

**A:** Frontal view of a single confocal plane of a 24 hpf embryonic forebrain stained with DAPI (gray) and immunolabeled for ZO-1. **B:** Frontal view of a single confocal plane of a 24 hpf embryonic forebrain stained with DAPI (gray). Top panel: red asterisks indicate cells dividing along the ventricle. Lower panels: higher magnification of the same cells. Scale bars = 50 µm.

**Figure S4. Expression of *shha* and *nkx2.1a* in the hypothalamic region at 48 hpf.**

**A-B:** 48 hpf forebrain following *elavl3* and *shha* *in situ* hybridization in ventral (A) and lateral (B) views. **C-D:** 48 hpf forebrain following *elavl3* and *nkx2.1a* *in situ* hybridization in ventral (C) and lateral (D) views. The general expression pattern of *shha* and *nkx2.1a* is consistent with the earlier stages, which label the hypothalamic region forming a conical-shape boundary. Scale bars = 50 µm.

**Figure S5. Dynamic changes of *otpb* expression in the ORR during the development.**

Forebrain region following *otpb* in situ hybridization and DAPI staining (gray) at 24 (A), 30 (B) and 48 hpf (C). A single confocal plane of a lateral view (top panels) and the ventral view reconstructed using Z-projections of the lateral images (bottom panels). Corresponding section levels are indicated in yellow lines. At 24 hpf *otpb* expression is merely found in the caudal region of the optic recess (A), while at 30 hpf it is found in both rostral and caudal domains of the lateral edge of the ORR (B). At 48 hpf the expression in the rostral region of the optic recess becomes more prominent (C). Scale bar = 50 µm.
