# Supplementary material for: Identification of the optic recess region as a morphogenetic entity in the zebrafish forebrain
Source: Sci Rep. 2015 Mar 4;5:8738. doi: 10.1038/srep08738 (PMC5390081; doi:10.1038/srep08738)
Supplement: Supplementary Information — Interactive PDF file demonstrating ventricular morphology [file srep08738-s2.pdf]

Identification of the optic recess region as a morphogenetic entity in the zebrafish forebrain

Pierre Affaticati[1*], Kei Yamamoto[1*], Barbara Rizzi[2], Charlotte Bureau[1], Nadine Peyriéras[2], Catherine Pasqualini[1], Michaël Demarque[1#], and Philippe Vernier[1#]

Unit Neurobiology & Development (UPR3294), Institute of Neurobiology Alfred Fessard, CNRS; Avenue de la Terrasse, 91190 Gif-sur-Yvette, France

Supplementary material: interactive PDF file

Reference views : Dorsal Frontal Lateral Ventral

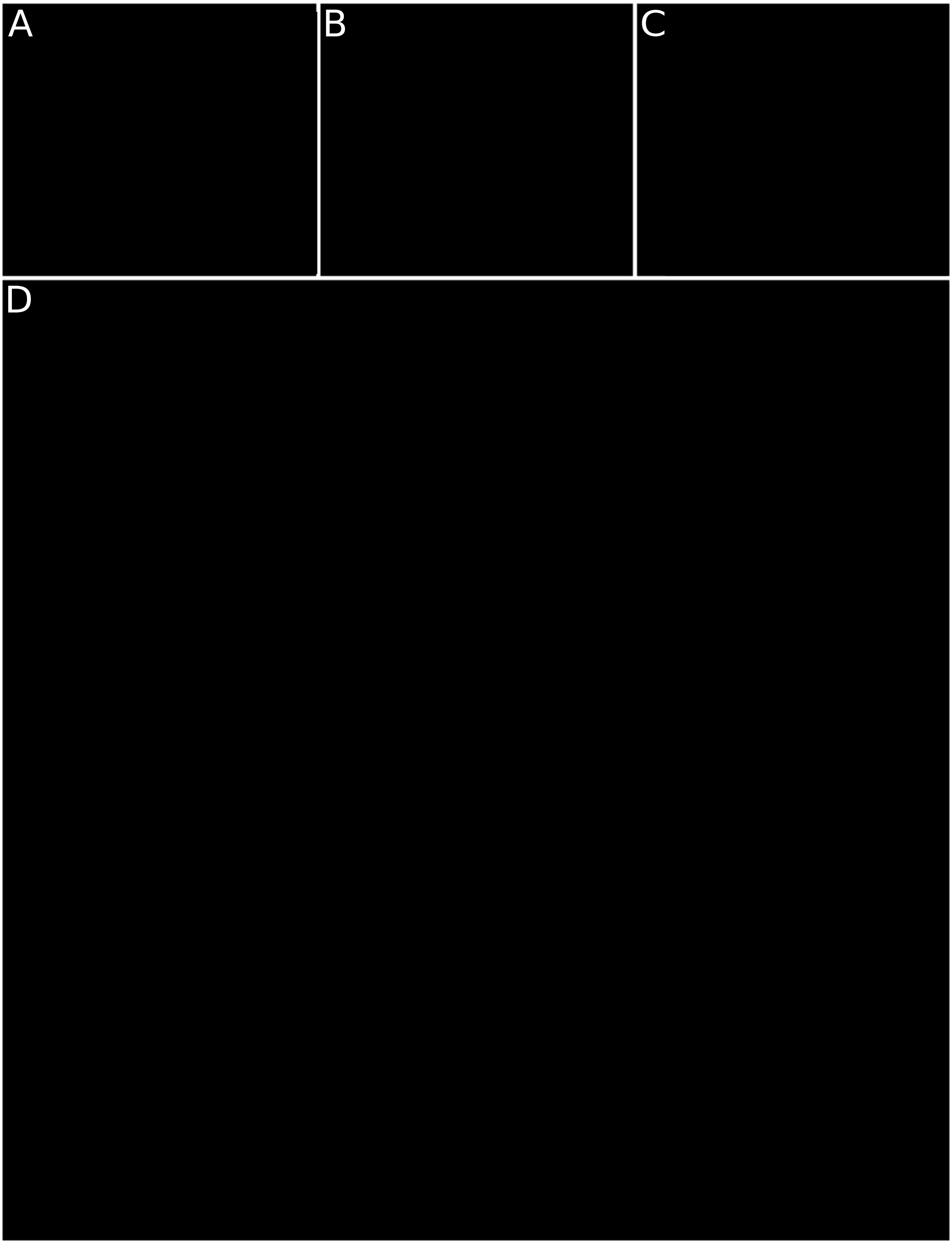

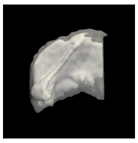

24hpf

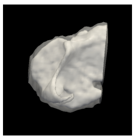

30hpf

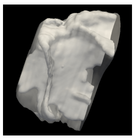

48hpf

Supplementary Material 1. Interactive PDF file demonstrating ventricular morphology.

3D interactive surface rendering representation of segmented ventricle (in opacity) and forebrain (in transparency) of zebrafish embryos at 24, 30, 48 hpf. Each figure can be rotated, zoomed and translated independently. **A-C:** Dorsal, frontal, lateral, ventral views at 24 (A), 30 (B), 48 (C) hpf. Clickable buttons allow simultaneously changing the views. **D:** Same images as in A-C at higher magnification, with lower buttons for choosing the stage to be displayed. On the bottom left is shown the reference of the original confocal image. Mouse left-click to rotate, right click to zoom in and out, both to translate. Abbreviations: Hyp: hypothalamus, ORR: optic recess region, Tel: telencephalon.
